# Participation in Communities of Women Scientists in Central America: Implications From the Science Diplomacy Perspective

**DOI:** 10.3389/frma.2021.661508

**Published:** 2021-07-12

**Authors:** Kleinsy Bonilla, Johana Cabrera, Camila Calles-Minero, Ivonne Torres-Atencio, Karina Aquino, Deysi Renderos, Margarita Alonzo

**Affiliations:** ^1^ Department of Science and Technology Policy, Institute of Geosciences, University of Campinas, Sao Paulo, Brazil; ^2^ Instituto para el Desarollo de la Educación Superior en Guatemala (INDESGUA), Guatemala City, Guatemala; ^3^ Department of Psychology, Faculty of Humanities, University of Santiago, Santiago, Chile; ^4^ Direction of Research, Technology University of El Salvador, San Salvador, El Salvador; ^5^ Department of Pharmacology, Faculty of Medicine, University of Panama, Panama City, Panama; ^6^ Instituto de Investigaciones Científicas y Servicios de Alta Tecnología, Panamá City, Panama; ^7^ DiploCientífica Science Diplomacy Network in Latin America and The Caribbean, Tegucigalpa, Honduras; ^8^ Innovation and Technology Transfer Office, University of El Salvador, San Salvador, El Salvador; ^9^ Ministry of Economic Affairs, Guatemala City, Guatemala

**Keywords:** women research communities, women in STEM, science diplomacy, Central America, Latin America, scientific diaspora, OWSD, women science networks

## Abstract

The experience of building and participating in women scientists’ communities in Central America is a multi-layered topic worthy of study. Understanding the dynamics of these women’s groups, associations, and other forms of collective participation, could assist in shedding light on why women are typically under-represented in Science, Technology, Engineering, and Mathematics (STEM) research in countries within this region. The objectives of this study are (i) to explore the experiences of participation in communities of women scientists in Guatemala, El Salvador, Honduras and Panama, and (ii) to systematize the challenges and opportunities derived from such activities. Additionally, this work elaborates on some best practices from the Science Diplomacy (SD) perspective, which could provide a helpful framework to encourage these types of collective participatory communities. The qualitative research methodology was based on the collection of primary data from semi-structured interviews and responses to an online survey sent out to Central American women scientists. The findings of this study revealed few cases of community building experiences among women scientists within the studied countries. Evidence also showed the emergence of shared patterns in terms of barriers and disincentives to participating in such communities. Meanwhile, data collected from the few existing community groups is used to identify successful incentives and motivations. The analysis of the collected data offered relevant implications for Science Diplomacy. Most respondents referred to the Organization of Women in Science for the Developing World (OWSD) as one of the main organizations that can impact and further Science Diplomacy. This organization promotes international engagement and networking among women scientists from developing countries across regions and this article shows how this has been used to foster women science community building in Central America. Exploring similar practices in-depth may offer opportunities to overcome traditional barriers and build further gender equality in science in Central America.

## Introduction

Central America represents an ideal geographical locus for examining the building of women scientists’ communities, as well as, the dynamics of participation within those groups. Guatemala, El Salvador, Honduras and Nicaragua have been categorized as scientifically lagging countries (Wagner et al., 2001; IDB Inter-American Development Bank, 2010; TWAS, 2020). All these countries, including Costa Rica and Panamá, have recorded inadequate research capacity (Padilla-Perez and Gaudin, 2014), when compared with global accepted standards. At a historic moment in which knowledge economies are ever-more powerful, these societies struggle to allocate the critically needed resources to build science and technology capabilities. Pressing problems such as acute poverty, structural inequality, political instability, and precarious access to basic services such as health and education, require the immediate attention of leaders and inevitably science and technology are relegated to a marginal position on the public agenda. In this context, the participation of women in research and scientific activities has been restricted by structural barriers which have diminished their ability to access career opportunities in STEM; furthermore, the possibilities for engaging in community building and participation are limited. In this context, since 1993 OWSD has provided a platform for networking and community building for women scientists living within developing countries, as well as those nationals from such countries but residing abroad (OWSD, 2020; Quadrio-Curzio et al., 2020). Reaching out to the scientific female diaspora has been a key feature of this networking, together with encouraged exchanges and collaborative processes among women from different parts of the world.

Regions such as Africa, the Middle East and South Asia have shown important progress with growing membership and active organization in the form of National Chapters. However, Latin America has made advances at a slower pace. This has been made more evident in the case of Central American countries; despite the 27 years that OWSD had been encouraging membership and participation in multiple activities, no national chapters were established within Central American countries until 2020. However, in just one year, Guatemala and Honduras national chapters were established, and El Salvador and Panamá began engaging in the process. With this in mind, it is evident that exploring the experiences of participation in communities of women in science in Central America may provide insights for other marginalized scientific groups. A National Chapter of OWSD consists in a community organized by women scientists national of a given country from the developing world (including those living within the territory or abroad) with the objective of promoting women’s participation in science and technology, scientific leadership, and scientific decision-making, both at the national and regional levels (OWSD, 2020). The processes for establishment requires a minimum of 20 members with the existence of a local host institution. Once an OWSD National Chapter is established, its members “carry out strategic activities according to priorities they identify within their own countries, including outreach to schools and the public” (Quadrio-Curzio et al., 2020:6) and also, focused in seeking career opportunities in benefit of its members.

Community building and community participation, for the purpose of this study, are understood as the various schemes in which women in science participate with a sense of group belonging with shared interests related to their research activities. These groups may be formal and institutionalized (i.e., science academies, associations, foundations, and NGOs) as well as informal groups (i.e., online platforms, collaborative groups, and discipline/subject-oriented networks). Nonetheless, a systematic and structured participation is required, which leaves out episodic group formations, such as those organized around a particular event or social group, that fades away once the purpose of their creation is achieved. The reviewed literature reports that most such scientific societies in Guatemala, El Salvador, Honduras and Panamá are male-oriented (Calles-Minero, 2010; WEF, 2020). Socially assigned roles and institutional gender-based biased have resulted a concentration of research STEM-related career opportunities for men. In consequence, those fields are considered unsuitable for women in the social context (IDB Inter-American Development Bank, 2010; Calles-Minero, 2013; Fernandez-Poluch et al., 2016). Moreover, the absence of specific policies and practices addressing gender inequalities in science and the representation of women in decision-making positions, as well as, insufficient efforts to create networks among female scholars and researchers have resulted in decades of isolated and insufficient women scientific community building experiences.

In this study, the researchers explored the possible root-cause for such outcomes, considering barriers and obstacles at individual, institutional, economic and societal levels through the collection and analysis of primary and secondary qualitative data. The incentives and disincentives experienced in Central America by women scientists in building and participating in science-related communities are highlighted. The conceptual frame offered by SD provides a special focus on the synergies presented by international engagement and the implications of such cross border exposure to enable community building among female researchers.

## Participation of Women Scientists in Community Building From the Science Diplomacy Perspective

Central American countries have relied for several decades on international support to construct their science and technology capacities (Bonilla and Kwak, 2014; Bonilla and Kwak, 2015; Bonilla et al., 2019), in this context SD studies may provide a helpful background to analyze science and technology relevant issues, such as the one covered in this research. SD is understood as a series of practices at the intersection of science, innovation and technology, and foreign policy to address common international issues (The Madrid Declaration on Science Diplomacy, 2019) and it aims at fostering international scientific collaborations among nations to address common problems, and to build constructive international partnerships. These practices can help to address global challenges, promote understanding, and increase influence and prosperity. In this view, scientific networks play an important role as a vehicle for sustainable development, cohesion and international relations (Fedoroff, 2009; Linkov et al., 2016; Balakrishnan, 2018; Mauduit and Gual-Soler, 2020).

Balakrishnan (2018), explains that SD can usefully be applied to the role of science, technology and innovation in three dimensions: 1) Scientific advice and inputs into foreign policy making (science in diplomacy) 2) Promoting international science cooperation (diplomacy for science), and 3) Using science cooperation to improve relations between countries (science for diplomacy). The second dimension of this proposed framework provides scope to this analysis as it focuses mainly on the facilitation of international scientific collaborations and the overall goal is to benefit from international science and technology resources in order to improve the national capacity; also, to build up joint partnership projects. (Melchor et al., 2020). Van Langenhove (2016) states that one of the components of SD, regards when groups are organized not only in disciplinary and epistemic communities but also in advocacy networks. SD is interdisciplinary and closely involved in bridging the gap between scientific workforce and diplomacy. Rhoten and Pfirman (2007) describe that the interdisciplinary nature of scientific networks may present junior women in science with valuable tools to overcome the structural and cultural obstacles of mainstream androcentric science. They also suggest that women are well positioned to make major advances in interdisciplinary research as they may integrate across fields and approaches, team-orientations, and be committed to connecting their research with societal concerns. The scientific debate on gender aspects in research systems has focused primarily on the overrepresentation of male academics, the productivity gap and issues of gender discrimination (Araujo et al., 2017). Furthermore, Rhoten and Pfirman (2007) state that the isolation of women is presently still problematic due to the historic overrepresentation of men in academic institutes, which constantly promotes the masculine culture. This asymmetry correction involves a complete stakeholder engagement process, including government support to create spaces and foster the participation of women in specialized communities that focus on specific aspects of collaboration. According to OWSD when women are included as both participants in scientific research and as beneficiaries of scientific research the impact on children, elderly and local communities will be directly positive and highly effective (OWSD, 2020). However, the global emergence of the COVID pandemic has delayed and even produced setbacks in the reduction of gender imbalances. While researchers might have harnessed improvements in telecommuting during the lockdown periods to focus on data analysis and publication writing, the gender bias intensified (Lopez-Verges et al., 2021; Ribarovska, et al., 2021).

It is well known that scientific research can lead to research outputs that could potentially solve many problems faced by developing countries. Although their scientific contexts still deal with diverse challenges, including the insufficient participation of women. The National Research Council (2012) states that one of the main challenges in the promotion of gender equity in science is the lack of a unified voice to speak on behalf of women’s specific needs to use science and effectively communicate with broader sectors of society. As for the link between gender, community participation and science diplomacy, a recent growing awareness has been raised in the literature (Cohen Miller et al., 2020; Quadrio-Curzio et al., 2020; Sharma et al., 2020), including the significance of inclusiveness and networking as principles for SD (Aukes et al., 2021) and the observation of a collaborative bottom-up approach (Moreno et al., 2017). At the regional level UNESCO (2020) recommends creating transnational scientific networks in order to strengthen the national system in less developed S&I contexts and foster “brain circulation.” In the same sense, the United Nations Economic Commission for Latin America, and the Caribbean (ECLAC, 2013) recognizes the importance of coordination for research across borders. Such practice could improve the dissemination of knowledge in those countries with shared interests in order to create a strong international network of scientists and evade the duplication of efforts. Thus, collective efforts to tackle regional challenges among science communities are meant to fix issues such as the distribution of public funds for scientific projects. Literature focused in Central America, also deals with other issues relevant to the formation and accumulation of the scientific workforce by means of engaging in international cooperation (Bonilla and Kwak, 2014; Bonilla and Kwak, 2015; Bonilla et al., 2018; Bonilla et al., 2019). Therefore, the Central American Integration System (SICA, 2020) may use SD practices by adopting partnership agreements with multiple stakeholders. However, despite the importance endorsed in building science communities in the region it has remained unclear if the participation of women in the research arena may result in major advances in interdisciplinary research.

## Materials and Methods

This study was carried out using a qualitative methodology in which three types of sources were used to collect data from women scientists in various fields in the participating Central American countries. Firstly, a “desk review” was undertaken to collect secondary data from reports, official documents, registries, and digital archives (Academia Guatemalteca de Ciencias Médicas, Físicas y Naturales, 2020; Ciencia en Panamá, 2020; SENACYT, 2020a; SENACYT, 2020b; SENACYT, 2020c).

Second, primary data was collected through semi-structured interviews with 43 women scientists. In order to ensure that principles of inclusion/exclusion were adhered to, those interviewed needed to fulfill each of three criteria: 1) women scientists with origin from one of the selected Central American countries (Origin), 2) accomplished researcher with recognition beyond her immediate research circles of influence (Prominence), and 3) experience in community building or community participation (Experience). A snowball and referral strategy was followed to build a robust list of potential participants. Diversity was sought in terms of representativeness by area of knowledge and stage in career development (early, mid-career and established) as described in Table 1.

**TABLE 1:** Criteria – selection of key participants—semi-structured interviews.

Criteria	Description	Operationalization
Experience	Experience in community building or participation, networking, groups of scientists	Reporting experience in building and/or participating
Trajectory	Procure diversity in the representation of career development stages of the interviewees (early, mid-established career)	Years since completion of graduate studies. Early <10 years, mid +10 years but no management positions or group coordinators. Established +15 years in addition to management or research group coordination positions
Prominence	Influence beyond her immediate field of work, preferably with national/international exposure	Publication, local or international award winning, participation in national and international activities in the scientific field
Field of Expertize	Diverse fields of knowledge (i.e., natural sciences, health, earth science, social sciences, physics, and engineering sciences)	All fields of knowledge were considered, including social, natural and engineering sciences

Lastly, an online survey was designed and implemented for data collection. The online survey was sent to all registered members of the OWSD National Chapters of Guatemala (340), Honduras (57), El Salvador (60) and Panamá (2) at the time of the study. The total membership list was built based on public records as for December, 2020 on the OWSD international platform. The survey was opened for responses from November 3rd to 15th, 2020 and received a total of 175 responses.

From the application of semi-structured interviews nearly 60 h of audio material was collected. Each interview session had an average duration of 45 min. From a preliminary list of 55 potential key respondents with curriculum vitae that applied to the selection criteria, a total of 43 interviews (see Table 2) were effectively completed following a strict set of criteria (see Table 1), using various online platforms. For example, using Google-meets Zoom, WhatsApp calls, and Microsoft Teams.

**TABLE 2:** Key respondents: semi-structured interviews.

Cod	Country of origin	Stage in career development	Research area
GT1	Guatemala	Mid-career	Food Sciences (Nutrition)
GT2	Guatemala	Established-career	Social Sciences (Sociology)
GT3	Guatemala	Mid-career	Social Sciences (Interdisciplinary)
GT4	Guatemala	Mid-career	Health Sciences (Geriatrics)
GT5	Guatemala	Mid-career	Social Sciences (Statistics)
GT6	Guatemala	Early career	Earth Sciences (Limnology)
GT7	Guatemala	Established-career	Social Sciences (Psychology)
GT8	Guatemala	Established-career	Chemical Sciences
GT9	Guatemala	Mid-career	Social Sciences (Anthropology)
GT10	Guatemala	Mid-career	Social Sciences (STI Policy)
GT11	Guatemala	Early career	Natural Sciences (Toxicology)
GT12	Guatemala	Established-career	Life Sciences (Entomology)
GT13	Guatemala	Established-career	Food and Nutritional Sciences (Nutrition)
GT14	Guatemala	Early career	Engineering Sciences (Nanotechnology)
GT15	Guatemala	Established-career	Agriculture Sciences (Virology and Horticulture)
GT16	Guatemala	Established-career	Life Sciences (Biotechnology, Microbiology)
GT17	Guatemala	Established-career	Health Sciences (Pharmacology)
GT18	Guatemala	Established-career	Interdisciplinary (Women in higher education)
GT19	Guatemala	Established-career	Life Sciences (Entomology)
HN3	Honduras	Established-career	Natural Science (Biology), Engineering science (Environmental Engineering)
HN6	Honduras	Mid-career	Natural Sciences (Elemental particle physics)
HN8	Honduras	Established-career	Health Science (Microbiology, Clinical Chemistry)
HN9	Honduras	Established-career	Social Sciences (Economist), transpacific relations
HN10	Honduras	Established-career	Engineering Sciences (Agronomist, Economy)
HN12	Honduras	Established-career	Natural Sciences (biology, Genetics)
HN13	Honduras	Established-career	Engineering Sciences
HN14	Honduras	Established-career	Natural Sciences (Neurology, Neurosciences)
SV1	El Salvador	Established-career	Social Sciences (International Relations)
SV2	El Salvador	Established-career	Social Sciences (Sociology, Education)
SV3	El Salvador	Established-career	Social Sciences (Economy, Rural development)
SV4	El Salvador	Established-career	Natural Sciences (Chemistry)
SV5	El Salvador	Established-career	Social Sciences (Philosophy, Legal Sciences)
SV6	El Salvador	Established-areer	Health Sciences (Medicine, Cardiology)
SV7	El Salvador	Established-career	Natural Sciences (Chemistry, Physical, Geology)
SV8	El Salvador	Established-career	Social Sciences (Sociology)
PN1	Panamá	Established-career	Natural Sciences (Neurology, neurosciences)
PN2	Panamá	Established-career	Health Sciences (Public Health)
PN3	Panamá	Established-career	Natural Sciences (Biology, Molecular biology)
PN4	Panamá	Mid-career	Natural Sciences (Biology, Virology)
PN5	Panamá	Early career	Natural Sciences (Neurology, neurosciences)
PN6	Panamá	Early career	Social Sciences (Anthropology)
PN7	Panamá	Established-career	Social Sciences (History)
PN8	Panamá	Early career	Natural Sciences (Chemistry)

N = 43.

The interviews were transcribed into text files and were codified and analyzed to determine patterns, trends, common content, and contrasting points of view. As for the Online survey, a total of 175 responses were received from Guatemala, El Salvador, Honduras, and Panamá with a resulting representation of 73,7, 10,3, 14,9, and 1,1%, respectively.

The compliance of ethics research guidelines was overseen by the Ethics Committee of the Technology University of el Salvador. All participants in the study provided informed consent to participate in the study and to publish the results, in accordance with national regulations and institutional requirements.

## Discussion and Findings

This section discusses data collected from the three qualitative methods as mentioned in *Discussion and Findings*. In order to provide structure and organization, five subsections are presented in this section. Where direct quotations were used, they have been categorized in order to facilitate the reading and respect the privacy of the participants.

### Experiences in Building Communities of Women Scientists and Researchers

In general, community building among scientists is not widely spread in Central America. Instead, initial efforts are identified in the creation of networks and other groups of researchers, including both men and women. Most of the interviewees responded that they had taken part in some form of collective participation in their countries of origin, or in international networking platforms. Overall the respondents repeatedly emphasized that the networking experience was an emerging practice, still vastly unexplored.

In Guatemala, the scientists network most mentioned was the International Network of Science, Technology and Innovation (RedCTI)^1^, which was founded in 2005. This organization has accumulated over 190 Guatemalan scientists residing within both the national and international territories. It is worth mentioning that the vast majority of members of this organization (71%) are men, with women members at 29%. The second most cited organization was the Guatemala Academy of Medical, Physical and Natural Sciences (AcaCienciasGt)^2^ which was founded in 1945. This organization is restricted to certain fields of knowledge. As of June 2020, the Academy had a total of 76 members and only 23% are women. This figures are worth noting as science academies in Latin American countries generally have a higher representation of women with 17% compared to the global indicator with a representation of women stall at 12% (IAP the InterAcademy Partnership, 2016:25).

In El Salvador the most mentioned networking organization was the Network of Salvadoran Scientists REDISAL^3^. However, this is a scientific registry organization and not a network of scientists. It includes a directory of researchers from El Salvador and as of 2019 a total of 1,035 scientists were registered, 38% of whom are women. El Salvador has not yet established a national academy of science.

Interviewees from Honduras, mentioned two main communities of scientists in their country: The Honduras National Academy of Sciences, founded in 1983, with 34 members in 2016. From them 5 were women, equivalent to a 17% (IAP the InterAcademy Partnership, 2016), and *Honduras Global* ^4^, an international network of “accomplished Hondurans”, including scientists and specialists from diverse disciplines. This network was formed in 2011 and currently reports having 54 affiliated members. This group does not have available data disaggregated by sex.

In Panama the most well-known organization for scientists is the Panamanian Association for the Advancement of Science (APANAC)^5^ a private non-profit entity whose mission is to promote science and technology to build the basis of national development. It was founded on January 4, 1985, and has 88 members as of 2020. The second most-mentioned organization was Science in Panamá^6^ a network of researchers formed in May 2016 which focuses on communicating science and seeking greater support from the civil society and decision makers within Panama, in order to increase the resources available for science and technology. This organization has about 150 members and provides a platform for scientific discussion, advice and promotion of the pressing challenges affecting the country.

The number of communities of women scientists in Central American countries is very low and there is extremely limited (nearly non-existent) support offered to such communities by institutions or local entities. Those few communities that are established for women scientists are short-lived (with few exceptions). The sustainability of their existence and activities appears to be dependent on individual efforts, including difficulties in transitioning leaderships, and communication difficulties between scientists from different generations and at different career levels. Once women in leading positions within women science communities retire several of the procedures and structures they have put in place may well close down altogether.

With regards to national communities exclusively formed by women in science, only a few were identified. In Guatemala, the most well-known community of women scientists is the Association of Guatemalan Women Scientists (ADEMCIT)^7^ founded in 2000 and still active. In El Salvador the Association of University Women (AMUS^8^) which has been a reference of women’s participation in higher education in the country since its foundation in 1952. Although this community has broader objectives beyond science and research it is also focused on education and training advancement for Salvadorian women. The second most-mentioned organization in El Salvador was the Network of Women Leaders in Higher Education (Red LIES^9^) which was launched in 2017, including ten universities committed to promote gender balance in academia, and is funded by the United States Agency for International Development. In Panama a number of organizations have promoted the participation of women in research communities such as the Feminist Women movement within *Ciencia en Panama* and APANAC, although they are not exclusively formed, nor focused on women. The Panama Smithsonian Tropical Research institute was also mentioned since it has promoted some initiatives specifically for women. Lastly, in Honduras there are no registries of communities of women scientists at the time of this study. It is worth noting that instead, the international Organization of Women in Science for the Developing World (OWSD) has registered members in all Central American countries. During 2020, this community established National Chapters in Guatemala^10^ and Honduras^11^, while El Salvador and Panama are still in the process of joining. Again, this is a global organization with national sections.

### Barriers and Obstacles to Participate in Communities of Women Scientists in Central America

In both the interviews and surveys, respondents elaborated on the numerous barriers and obstacles to their participation in scientific communities. The data was organized for analysis and discussion based on the instrument for the semi-structured interview (available in the Supplementary Materials), the survey added content to the categories. Table 3 below summarizes the main findings presented in this subsection. Excerpts of interviews are also included when relevant to enhance and elaborate specific issues.

**TABLE 3:** Barriers to building and participating in scientific communities for women in Central America.

Individual/personal barriers	Related to (psychological, cultural, gender-conditioned) personal interest, aptitude, persistence, lack of resources, perception of lack of family and social support, psychological insecurity, poor health, and low self-esteem
Family-related barriers	Related to childcare, care responsibilities for convalescent/disabled relatives, families not conducive to female education and empowerment
Institutional barriers	Related to unsupportive or discriminatory rules and structures and their implementation, lack of access to educational programs and educational and science curriculums of quality, lack of support to the family structure, lack of incentives, lack of institutional wellbeing dynamics in general—culture of isolation and non-cooperation, lack of education and training of soft skills, e.g., leadership for women
Economic barriers	Inadequate, outdated and unsupportive legal framework, lack of economic resources for science at all levels
Social barriers	Scarce relevance given to science at all levels, direct, and indirect violence against women, underrepresentation of women in decision making processes at all levels

#### Personal Barriers

The personal barriers are identified as issues at the individual level, negatively affecting the likelihood that Central American women scientists will take part in community building. In this context, the responses include: lack of time, multiple responsibilities, psychological perceptions, and gender roles. Respondents also cited multiple responsibilities which impinged on their freedom to participate in extra-curricular or career-development activities not offered by the institution where they work and study.

The multiplicity of activities in which women have to fulfill responsibilities places enormous pressures which limit our available time. With very tight schedules few of us remain interested in creating or participating in associations. Also, many female scientists are still unaware of the benefits that networks and communities can offer to their personal and professional growth as women and as scientists. Rather, network participation seems like a way to socialize, very superficial, not really worthy of our scarce time (Participant HN12).

I believe that we live in societies in which the multiple roles we are expected to play, put a burden on our shoulders which it is almost impossible to fulfill. Social responsibilities for women in the private sphere affect our careers (Participant GT3).

I don’t know of any groups or networks of women scientists in El Salvador. [I think] there is no interest. It has not occurred to anyone to promote this. What benefit will it bring? I would say both personal and institutional. If it is possible to combine these two things, it is fabulous. The economic part, because developing activities like these usually needs a budget and they want to give very little (Participant SV7).

#### Family Related Barriers

Family barriers must be taken into account both for those women scientists who have chosen to form a nuclear family with life partners and children, as well as for those who may have chosen a different path yet still have extended family-related responsibilities. It has been documented that the care of vulnerable and dependent family members such as elderly relatives and convalescent or disabled relatives generally falls on women (Hernández and Lara, 2015). Public policies are needed in each country to provide the conditions to enable a balance between work and family-related responsibilities for women scientists.

Family responsibilities frequently represent disadvantages for women to take part in groups or networking. In El Salvador, women start their families during university education, this brings complications as they are expected to be educated and trained to become competent researchers, and at the same time take care of their families (Participant SV6).

The absence of public policies that allow women to make their family life compatible with their work life. The traditional role expected of women in our society is burdensome in this sense. The multiple activities in which women are involved, do not give them any spare time in their busy schedules to be a part of building communities of women in science (Participant GT5).

#### Institutional Barriers

Institutions have an important role to play in promoting and supporting the participation of women in scientific activities through the implementation of gender-parity legal structures, high quality education (including soft skills) and women empowerment which is elemental for the construction of scientific communities. The institutional context includes any practice that may facilitate or hinder the construction of scientific communities, such as the structure, management style, and type of leadership. In general, from the answers received, it seems that, in Central America since there is little understanding at high level of the benefits to women of being members of a scientific community, with the result that institutions provide no resources or opportunities for such communities to be established. It is necessary to promote scientific research of high quality and encourage appropriate collaborations among individuals, groups, and leadership persons in order to initiate and enable participation within institutes. Making people aware of the importance and potential of networks must be an institutional responsibility.

In universities, the gender inequality dynamic between men and women is clearly evident and this limits the potential opportunities for professional and personal growth for women in science. This is demonstrated in the disproportionate representation of women in positions of authority within different institutions. This is particularly contradictory as Latin America shows higher representation of women in research compared to other regions (Lemarchand, 2010, pp 56–61), yet women are disadvantaged in terms of holding positions.

The gender gap in higher education is striking. When we analyze the number of department directors and academic units the imbalance is appalling! The number of men in leading positions is disproportionally large, the number of women is dismal (Participant SV6).

I think of the university; patriarchy has shown a strong resistance to gender issues. The situation has reached levels of misogyny, and a deep invisibility of women’s work (Participant SV2).

I think we have been evolving slowly and with differences between countries, some have acquired more rights and more support but there is still no comprehensive system (Participant HN9).

According to various interviewees, the structure of the institutions does not favor the development of science careers for women. There are no clear parameters for conducting research within organizations. This phenomenon is perceived in universities and research centers where researchers combine their teaching and administrative responsibilities. Many women are science practitioners within higher education institutions in Central America; however, their work goes largely unrecognized:

Institutional recognition of women’s participation in scientific communities is required. This issue has been addressed in the diagnostic report of women in science in Panama in 2018, Men [who usually make decisions in academia] have a bias about the role and value of women in science, they do not see us as peers, and this leads to the “scissor effect.” At the moment women begin their careers as scientists, they also start their families, but institutions do not accommodate this, for men this is not an obstacle, yet women must compete at the same level as their peers. Institutions must create conditions that correct this bias and support the construction of scientific communities (Participant PN3).

In order for women scientists to have well-balanced family and professional lives an institutional effort is important and needed. Therefore, institutions need to value the contribution of women scientists and fairly evaluate and empower women’s engagement in science:

Academic and research institutions here [in Guatemala] work like islands, isolated from each other, there are not many opportunities for interaction among female scientists and neither are there forums where we can exchange ideas and engage in initiatives of collective construction. I hope that the explosive growth in virtual communication and platforms will open many doors. For now, it is really hard to meet other women who share research interests. We work in very isolated conditions, even within the same institution. Where I work we are small islands within the same institution (Participant GT15).

Although the figures show great gaps between men and women in terms of academic titles, distribution of positions of power, and access to scientific careers, this reality is overlooked within institutions. Indeed, the narratives of respondents recommend raising and building awareness in women and encouraging them to gain ground:

I find it very disturbing how institutions accept and normalize these tremendous differences in the representation of men and women among authorities, professors and researchers. What is more, the few women who manage to stay in academia do not receive sufficient stimulus and support from institutions. We continue in science because we love what we do, imagine how much more we could achieve in better conditions (Participant SV6).

These efforts can be oriented toward the construction of scientific communities of women in which scientific production is shown with equity and on equal terms.

I know of attempts [at community building], and I have participated in attempts. These efforts to consolidate science collectively, if they do not have a central foothold that makes scientists form nuclei, will not be achieved, just getting together is not enough. If [such communities] are not initiated by government, they are very difficult to sustain (Participant SV4).

#### Economic Barriers

Interviewees stressed how essential funding is to starting a successful career in science. However, job instability for women undertaking scientific research living in Central America represents a serious obstacle to participation in collective activities. The women interviewed often describe working under unstable conditions, juggling between one or two jobs in order to make ends meet. Women scholars who have secured a full-time job in academia already struggle to fulfill their administrative and teaching duties, since research activities are not recognized as part of their full-time job descriptions (this frequently happen also for men).

In my university we limit ourselves more to administrative work in our desks, I do engage in field research work but many times we have to use our own funds to do things, so that is very limiting. Speaking specifically as a woman, then perhaps one difficulty has been that the environment is dominated by men, so the way of working is sometimes very dominated by them, and the environment is very competitive too ... it’s both good and bad. Sometimes it makes it difficult to accept a woman in a working group and well, sometimes we have to deal with difficult people, not all, but some ... but there are always so many men and women, not only men. So that has been the most difficult as a woman (Participant HN13).

Women interviewees expressed that despite having obtained scientific qualifications and skills they have found it difficult to develop their careers. Scientific development does not have concrete support in the countries under study.

The limitation of the country [Panama] is that we are few, and there is a lack of infrastructure and funds to be able to integrate, especially in terms of women scientists. It is a limitation as there is a lack of a system that allows adequate insertion. Although we are a small country, there is not enough interaction between scientists in the city and those who live in the provinces, the way the city is conceived does not facilitate activities after work since time is wasted on returning home and takes away the opportunity for connectivity (Participant PN4).

There are many issues to work on in Guatemala, the main limitation is job stability. That always worried me and I am reaching the age that I should think about retirement, but it has not always been possible to obtain funds for my work. Local funds are very scarce, and the type of grants that can be obtained through the national system are insufficient to support a meaningful and world-standard research project (Participant GT12).

Despite scientific job instability in Central American institutions the narratives of women interviewed suggested faith in the possibility of strengthening their scientific work with the support of private companies and the government. However, a positive vision from these actors (private firms and public institutions) is needed to promote and support women scientists’ research productivity.

Our employers and bosses are willing for us to form these networks but they see them as something secondary that has no real practical value, so the question is how do we motivate these institutions to give value to associations of this type? (Participant GT13)

Basco and Lavena (2019) confirm what was stressed by interviewees, that women’s participation in the labor market in science and technology moves from exclusion to horizontal and vertical segregation taking into account the different economic empowerment scenarios.

#### Social Barriers

The social conception of networks of and for women in science is that they are unnecessary or tainted with “extremism” on the part of those scientists who wish to build them in the Central American region. The gender bias in education is not yet understood and when communities are exclusively formed by women (not only women scientists) they tend to automatically labeled as “radical feminist” organizations, confirming deeply rooted misogynistic prejudices in male-oriented societies. It is important to re-evaluate the role of women in science and technology and to rewrite history to recover women’s or “feminine” traditions from oblivion. Despite having made notable contributions to the scientific-technological field, women are still not recognized for their contributions:

Science in general is not a priority in my country, [therefore] the role of women in science is an element even further down in the social priorities. There is a divorce between academia and gender in El Salvador. Feminist studies, women studies in any field, the gender scope is absent. Women in science is still a pending issue. Let’s say that it is a structural situation [barriers for women in science], that we can also take as an obstacle, in the sense that the development of science is very limited, including the social and natural sciences. The war greatly fractured the development of the sciences in El Salvador, in such a way that the university, I believe, still has not recovered from that, because communities, in general, have not been created, and much less so communities of women scientists, in any space, school or faculty. Because the conditions for this to happen did not exist. Who was going to coordinate the establishment of such communities from within the existing structures? As the gender perspective in academia has not and still is not considered relevant the few available studies have been carried out with the support of NGOs and therefore the scholarly community does not accept or validate them (Participant SV2).

The findings showed an established social role of household administration as one of the main barriers that women have to develop in the scientific world.

The lack of participation of Salvadoran women in groups, networks or communities of researchers has to do with the issue of gender roles and stereotypes. It is very common for men to meet after work hours until midnight, or even in their own homes, to dedicate themselves to socializing and taking part in groups. Meanwhile, women have to run around to reconcile professional work with domestic work, I think that limits us (Participant SV3).

Despite the social barriers, the survey participants viewed the creation of science-related with optimism, and recognized that these networks can yield opportunities to overcome social barriers. However, the efforts needed to build networks or include scientists must take into account the multiple challenges that affect women:

I think it is something very recent that these movements are beginning to take place, when I started my career they did not exist, they are still very few but I see that there is a movement that is interested in creating these research networks. I think we have to reach a critical mass of people who are working in research in the country to create these networks. Then having a volume of people who are working in the area it can make sense to form a collaborative network but if there are few people who are doing the work in the country it does not make much sense, that is why it is important that there are enough people working on the themes so that there are areas of common interest for the formation of these networks (Participant GT13).

It is necessary to educate about a gender perspective; even in Panama it is considered “radical” to be part of a group of women scientists. On the other hand, I have no evidence in this regard, but I assume that those of us who are currently part of this group do so because our positions are not at risk (for different reasons). It is up to us to strengthen scientific and academic institutions so that more women at all levels of professional careers can belong to communities of women scientists without this implying professional risk (Participant PN1).

Various societies in Central America have experienced significant violence in their recent history. In Guatemala and El Salvador, both countries were involved in long-lasting armed conflicts in the second half of the twentieth century: for 36 years (1996–1999) in Guatemala and 12 years in El Salvador (1979–1992). These traumatic episodes have affected the entire Central American region, destroying multiple layers of social tissue, and discouraging the formation of and participation in collective processes (Navas, 2018). Knowing this context is important as various respondents point out the constant discouragement to collaborate and form groups affects most generations of Central Americans. As a result, women face specific challenges regarding creating and developing scientific communities.

Despite the efforts of many women to demonstrate the value of scientific activities, they are still undervalued by Central American institutions. This situation was also referred to by interviewees, who indicated there is a tendency for institutions and governments not to value or strengthen scientific research in general, much less that carried out by women. However, women have made efforts to overcome these barriers, as we can see from the following narratives, which suggest that there is a need for systematic work to provide women with the necessary tools to dedicate themselves to science.

Women experience epistemic violence due to the androcentric nature of science and knowledge production. The system still doesn’t take women contributions to science seriously. If we review even at the level of decision-making spaces within the university itself or within the different spaces of life in Salvadoran society, it is in the hands of men; The structures in academia concede privileges and attribute to men qualities which women do not enjoy; but it is not easy at all, in fact in the economics school to which I belong there are 35 faculty members, and only four of them are women. And this is striking considering the perception that in the Social Sciences participation of women is perceived as higher and more active compared to engineering, natural sciences and STEM fields (Participant SV3).

In Panama, society does not see the need for women to build exclusive groups for them. We have low visibility, women are not seen as the experts they are, men have monopolized the public’s respect and attention. In general, women are not recognized as experts (Participant PN7).

### Benefits and Motivations to Participating in a Community of Women Scientists

**FIGURE 1:**
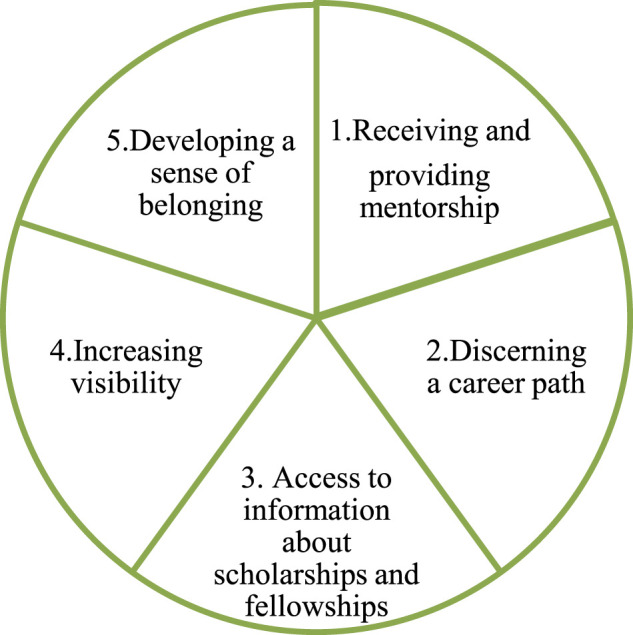
Benefits and motivations to participating in a community of women scientists.

Evidence suggests that a significant portion of Central American women scientists still overlook the numerous possibilities that group participation has to offer their career development. Nonetheless, when asked about the benefits (real or expected) of being part of a group or community of women scientists the participants provided the motivations shown in Figure 1:

#### Receiving and Providing Mentorship

In Central America mentoring has not been widely explored as a systematic practice among women scientists. However, several of the interviewees and respondents pointed out that providing and/or receiving mentoring is a key feature they seek when participating in networks or communities. (Oshinkale, 2019) provides a helpful definition of mentorship and the mentor-mentee relation:

“A mentorship is a relationship between two people where the individual with more experience, knowledge, and connections is able to pass along what they have learned to a more junior individual within a certain field. The more senior individual is the mentor, and the more junior individual is the mentee. The mentor benefits because they are able to lead the future generation in an area they care about and ensure that best practices are passed along; meanwhile, the mentee benefits because they have proven that they are ready to take the next step in their career and can receive the extra help needed to make that advancement” (Oshinkale, 2019: first paragraph).

Mentoring (formal or informal) is seen as a mechanism to strengthen women’s performance in various disciplines. In addition, interactions between women scientists in different stages of their careers enable broader understanding within science and lead to scientific collaborations. Mentoring also nurtures leadership and empowerment skills among women scientists. Most of the women with a consolidated career who participated in the study have been leaders in scientific initiatives in their countries. Some senior women also joined communities of women in science or became members of recognized scientific institutions. However, due to the time constraints, administrative work load and other duties, only a few have developed their careers as heads of their institutions or programs. Inequity persists throughout the different fields of the academy not just for research project selection, but also when it comes to publishing or applying for leading positions:

During my undergraduate and postgraduate, I tried to be surrounded by other women and actively participated in mentoring. This allowed me to become even more aware of the gender perspective in Public Health research (Participant PN2).

I benefitted from mentoring, rather informally probably because there were very few women at the beginning of my academic career. I see that after several years, the presence of women has increased in my field. I try to return the positive experience by being a mentor myself, it is also important to inspire the students and guide them. It is really helpful for girls to have female mentors (Participant PN7).

I think men have been successful in building ties between them, establishing a sort of mentorship in informal settings, compared to women. They push each other up, or when they are up, pull up the aspiring young male researchers. We should work to create our own communities (Participant GT1).

#### Discerning Career Development

Women in science, and scientists in general, face challenges when it comes to combining their scientific career with administrative or teaching positions. Research activities are still not included as core duties in the description of job positions in both public and private sectors. The lack of scientific research within these sectors is a culture that needs to change, the experiences expressed by the interviewees emphasize this challenge:

People in El Salvador get their [undergraduate] degree and then they stop there, if they find employment in a university, the truth is, there they stay, but there is no growth, there are not sustained interest in career development, there are no competitions, there are no incentives. In other countries, professors are encouraged to improve their knowledge, by being giving particular incentives (and salary raises) if they continue studying, if they participate in conferences, if they offer presentations, all of this is worth points in other universities. There is no involvement because if you are required to attend a conference you don’t have funds because the university does not pay (Participant SV2).

By joining our voices, we can be heard, we can grow stronger and faster in concerted paths. There are issues of egos as well, of mistrust, there may be so many reasons, sometimes we do not want to participate due to experiences in the past, that is why I think a few of collaboration between scientists can be beneficial (Participant HN14).

#### Access to Key Information About Scholarships, Training and Other Incentives

Central America has relied on international cooperation to educate and train its scientific force for decades. The underdevelopment of higher education systems and insufficient conditions to pursue graduate studies in the national territory (Bonilla and Kwak, 2014; Bonilla, and Kwak, 2015; Bonilla et al., 2018) has created a permanent search for scholarships, fellowships and financial funding to support studying abroad. Respondents expressed that among their main motivations for joining a network or a group is to have access to information about the availability of these opportunities to continue their education and training:

Progressively the institutions are taking on perspective of the gender-harsh context in Central America. At first the role of women scientists was not evident, but we have advanced at least symbolically with recognition through local awards such as the L’oreal- UNESCO Women in Science fellowships, which already has had four national editions (Participant PN2).

I obtained a scholarship to complete my graduate studies supported by a foreign government, otherwise I would not have been able to do so. After that experience, I realized there was an opportunity for me to encourage other Salvadoran women to apply for scholarships. That is why I joined various networks I could identify to pursue this purpose (Participant SV6).

#### Increasing Visibility, Exposure and Recognition

When women join communities they increase their exposure and the visibility of their contributions and career development. This exposure and recognition enables further impact in their societies as they act as role models for children and young girls. There is a staggering shortage of female figures engaged in science in the Central American. Nevertheless, various scientists interviewed pointed out that in their countries there is limited value and insufficient recognition for scientific activities and production.

The evolution of communication, and social networks, has played a part in motivating girls and young women, letting them know that there are other women scientists opening doors for them. The implementation of institutional measures for gender equality, which is being worked on by SENACYT [The National Secretariat of Science and Technology] has a committee, with regulations, and more can be done. Also the encouragement of networks to put women on the radar of public opinion (Participant PN4).

#### Sense of Belonging

Women scientists have just started to organize in a more systematic and widespread practice in Central America. As the number of women participating in research and science activities increases the opportunities to connect are yet to be further explored. The role of women pioneers in science in this region is valuable and fundamental in order to bridge gaps and disrupt working in isolation. They can overcome steep barriers and start communities from scratch and they can inspire an upcoming generation of scientists. Thus, the interaction between women in different stages of their careers may connect, share ideas, work together and have a sense of belonging with recognition.

#### Critical Perspective Toward Women Networks and Communities

It is important to acknowledge women in science that criticize this initiative and do not see the benefits of being part of a community of women. Some of them have indicated that when they became involved in research there were only a few scientists in the region and communication systems depended on meeting in person which entailed logistic hurdles, and the use of resources and effort. With the access to internet and mobile communication, relations between scientists from all over the world have been facilitated and interactions and building connections have become more accessible. Some women in science are still debating the benefits to invest time in communities as they indicate below. In some cases, networking and community building is perceived as a burden instead of a support mechanism for career development:

I feel conflicted when thinking about groups of only women and wondering if that is effective. I think that for Guatemala it is necessary to grow together in networks, men and women, supporting each other, I think that for the good of the development of science all of us should be represented (Participant GT11).

I have not actively engaged with communities of women scientists because I don’t see it as a priority, and the benefits are unclear. Funds are not given for this activity. This must be adjusted. Also, if we think about mentoring within networks we have to be precise about who will benefit from it, which population will be targeted, and it must be well planned to be sustainable (Participant PN8).

### Enablers and Disablers in Community Building Among Women Scientists

**FIGURE 2:**
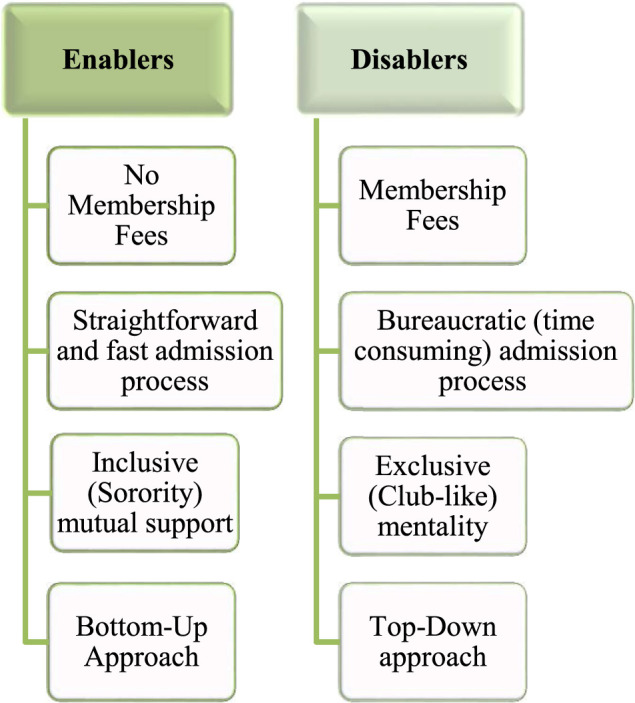
Enablers and disablers in community building among women scientists.

Considering the numerous barriers women scientists encounter either to construct or to participate in communities, it was important to explore the enablers and disablers of such participation. These are conditions, requirements or processes that could facilitate or further discourage women’s participation in groups. Figure 2 depicts the most often cited elements seen as facilitating or discouraging community participation.

#### Paid/Non-Paid Membership

Given the burden women in science experience in Central America in terms of job instability and lack of career development, the requirement to pay for membership was mentioned by various participants as discouraging them to take part in groups:

An indisposing requirement to belong to networks or groups is a paid membership. Honestly even when it is not much money, we cannot afford it. Specially in precarious employment contexts for women in science I guess, there have not been enough initiatives to build communities and not enough commitment, but I also think that many women who want to participate just cannot pay membership fees (Participant HN3).

#### Simple/Bureaucratic Entrance Process

Some existing scientific networks in Central America were characterized by various respondents as having a bureaucratic application process in which they had to submit several letters of recommendation, proofs of academic achievements in printed documents and that the opportunity to join was open once a year; subjecting the approval to join to a general assembly. In other cases, participants indicated that the application process was time-consuming and difficult to follow guidelines led to confusion during the application process.

To be honest, I felt a bit frustrated when trying to be active in a couple of groups of scientists in Guatemala. It took a long time to complete the paperwork to be accepted, and once I was part of the communities, they were too slow. Making decisions took a long time, in the sense of some directive members had almost veto powers (Participant GT1).

I applied for membership in 2018 through a “speedy process” because my profile was recognized with an award which gave me a green light to join the group of scientists. It was two years later that my membership was approved, I think it was too long (Participant GT11).

#### Inclusive/Exclusive Practices

Higher education in Central American societies is affected by inequality (Cox, 2010; Bonilla and Kwak, 2014). In fact, inequality is both deep and spread out over multiple dimensions from race and gender to unequal access to education, health and other services (Busso and Messina, 2020). In this context access to university studies has been restricted to traditionally privileged groups in urban areas. There are several layers of exclusion that also affect leadership in research groups and networks. These conditions need to be taken into account, as the trends behind community building between women scientists have been depicted as taking either one of two approaches: inclusive diverse organization and exclusive prestige-based groups.

Networks and communities of scientists too frequently are managed like clubs. You must take the initiative to reach a level with recognition to introduce yourself to those clubs. The way to do it is to have the endorsement that says “I would like to bring that person.” Volunteering and doing hard work may not be enough. You need connections to be accepted (Participant HN8).

In Guatemala there was an association of women in science, I think it was created in the 70s, unfortunately there was a very marked difference of social class, so I did not feel welcome. In addition, the areas of knowledge of the members were too confined around chemistry and pharmacy. Also, the leadership was very concentrated, rather than a horizontal, inclusive environment, decisions were taken by one or two members. Later, in the 90s, a group tried to give that association a spin, but they were unsuccessful (Participant GT2).

#### Bottom-Up/Top-Down Approach

Democracy for all citizens is still undeveloped in Central America. Most of the countries under study were affected by successive dictatorial military governments. This has affected social and political dynamics, the way citizens receive basic rights to education and the prevalence of centralized planning in public policies. This is relevant as the formation of national innovation systems in Central America has relied on the top-down approach (Padilla-Perez and Gaudin, 2014), and the formation of communities of scientists must be placed in this context. The answers provided by respondents suggest it would be beneficial to aim at organic organizational movement initiated from the grassroots (bottom-up), instead of promoting “compulsory” participation (top-down) based on official appointment.

In various cases of networks and groups we were appointed by authorities to represent our institution. I believe this diminishes motivation, we have interpreted this as an extra burden to our workload instead of an incentive (Participant SV5).

In my experience, scientific initiatives begin and are promoted by the government, the private sector does not get involved as it does not yield profit. We have left the responsibility to organize formal group organization of scientists to the government or public universities (Participant SV4).

### Science Diplomacy and Women Scientists Community Building in Scientifically Lagging Countries

The inspiration for women in science to organize and identify the dynamics and benefits in building and participating in communities has come from abroad. As has been discussed earlier, the first Central American women engaged in scientific research worked in isolation in a predominantly male environment. A number of participants expressed the positive role that science diplomacy has played and mentioned that more could be done to encourage and support groups and collaborations between women scientists in Central America.

#### Cases of Emerging Science Diplomacy Experience and Community Building in Central America

Remarkably, various participants referred to assorted emerging examples of science diplomacy practices linked to networking and community building recently implemented in Central America. The promotion of scientific collaboration in national and international schemes could aim to solve regional challenges with a multi-actor perspective among countries with similar challenges.

*Converciencia* is the annual meeting between Guatemalan scientists who work in research within and outside the country and broader sectors of the Guatemalan society. For twelve years SENACYT has promoted this activity with the vision of creating synergies between compatriots for the exchange of knowledge, experiences, good practices, and opportunity management and links that have an impact on the strengthening of a national scientific agenda. This event makes visible the quality and talent of the participating scientists and their commitment as Guatemalans, both residing in the country, and residing abroad, providing support from within their different disciplinary fields and contributing to the challenges and challenges of the country. In Guatemala we are in the process of integrating actions to address problems and challenges, and this activity allows us to foster spaces for interrelation that will help increase competitiveness (Participant GT11).

*Honduras Global* was founded in 2011, and aims to identify and connect Honduran and highly qualified worldwide scientists in order to promote the transfer of knowledge and promote innovation and scientific, technological and business development in Honduras. It is worth noting that our priority activities revolve around innovation, science, technology, and entrepreneurship. The initiative has had some support in terms of funds from the Honduran government that varies according to the administration. Also, it has support from local research entities, there has always been a very good exchange with universities in Honduras, mainly with UNAH [National University of Honduras], which in my view is a very important entity at the national level for certain careers, and we have had collaboration with private companies and it has been very fluid, but regarding your question about collaborating with multilateral organizations, this has been quite difficult to establish (Participant HN10).

In collaboration with the Gorgas Institute for Health Studies, in 2006 the Health Diplomacy program was developed together with the United States Department of Health, but it covered a collaboration with the entire Central American area, specifically focused on influenza. This experience was later transferred to the Center for Disease Control and expanded to HIV, in the latter they worked with countries such as Costa Rica and El Salvador. Other projects such as those developed with the University of South Florida were more aimed at high-risk and vulnerable communities such as indigenous regions (Participant PN2).

[In my university] we are part of the Superior council of Central American universities, and through this platform we are trying to improve the quality of researchers of scientists in Central America and so this could be called science diplomacy. But it seems to me that this is being led more by the universities than the government (Participant HN13).

#### Opportunities for International Engagement and Science Diplomacy Practices Addressing Community Building in Central America

In recent times, efforts have been made by international organizations to achieve science diplomacy practices in the regions under study. The aim is to open a window of opportunities that create scientific communities with a balanced gender perspective. For example, the General Secretariat of the Central American Integration System (SICA, 2020), together with the Central American Higher University Council (CSUCA) and the Commission for Development Scientist and Technologist of Central America and Panama (CTCAP), with the support of the International Development Research Center (IDRC) of Canada collaborated to promote regional scientific collaboration. In 2020 they launched a Regional Call for Projects for the Organization of Central American Networks and Communities for Scientific Exchange where researchers in the region were encouraged to submit proposals, and highlight regional priorities in the context of the COVID-19 pandemic.

(1) Central America integration

Placing gender imbalances, differences, and gaps as priority topics on the agendas of policy makers is critical in Central America and vital to their potential incorporation in national Science and Innovation proposals and programs.

(2) Mapping out the Central American women scientific diaspora, through robust directories

There are no consolidated directories of women scientists which include the Central America scientific diaspora. Informal networking through social media (Twitter, Facebook, Instagram) as tools to connect has been of interest in recent times. This topic was addressed by some of the interviewees:

[The OWSD national chapter in El Salvador] should map how many women do research and distinguish by area of knowledge. It would be valuable to increase the visibility of Salvadoran female researchers, so we can know them. The information must be more dynamic so that the benefits can be seen more and more participation. Academic women need spaces for development. I believe that this network has great power to fill real needs or disseminate the opportunities that exist. Communicate what we have and promote national events to meet the entire Salvadoran scientific community, including all those who reside abroad (Participant SV1).

I think that maybe this is the moment to create a scientific community of women in Honduras. Before, there were very few of us, the efforts made did not bear fruit, but right now, for example, in the physics department there are many girls studying abroad, engaged with scholarships, holding conferences, performing well in international collaborations. There are Salvadoran women in the United States, working with NASA, also in astrophysics, and in other countries. I think this is the right moment to consolidate or settle the foundations for a community (Participant SV7).

We need to highlight more women scientists in Honduras or those Hondurans in the scientific diaspora, make spotlights in the different scientific areas, and show who the women are behind these disciplines. Within the scope of the study of journalism, the person who interviews you must be a journalist who has training in science and technology. We need to involve the private sector, academia, public institutions and wider society (Participant HN8).

(3) Institutional Reform within the Ministries of Foreign Affairs

There is a gender gap in foreign policy in Central America. Generally, women are underrepresented in foreign policy circles and this is not new. The diplomatic circle also remains overwhelmingly male-dominated. Even in international negotiation events and activities promoted in Foreign Ministries of the region most speakers are men who represent their governments. Thus, there is a risk of missing important insights that women can incorporate to science diplomacy and community building with a gender balance.

When I observe the handling of my country’s foreign policy, we generally see men as delegates, as representatives of the Salvadoran State. For example, I observed the case of the Japanese cooperation, that is, if they do a lot of scientific research with my university, it will be with the Faculty of Agronomic Sciences, where we know that they are mostly men. I do not know from first source how many women are participating in this or how many women have the opportunity to participate in these exchanges in a personal capacity or as institutional representatives. There is still a huge gender gap (Participant SV3).

(4) Science Diplomacy and Policy Advice

The COVID19 pandemic has put scientific advice to the forefront in guiding and providing solutions. Various actors (including scientists) started focusing on how science should be taken into account to inform and advise decision makers in situations like COVID-19. The challenges are related to how scientists communicate the results of research findings and transmit the importance of these results to our wellbeing.

For me, having the opportunity to research, publish results, or found groups of researchers, has been a complete journey. My work is in the entire experience, not just doing research and publishing, which is the expectation. But taking the research to the next level has been very interesting, then being able to influence policies in programs by being part of the lobby, even before the national congress and before other entities has been a very good experience (Participant HN14).

(5) Science Diplomacy and studies about women’s careers

Science diplomacy could also have an impact on the production of further studies, materials and information about gender gaps in science in Central America.

Science diplomacy could contribute above all with studies of women scientists in Central America, then we could see the progress of gender in science around the world and have a better perspective. I heard of interesting initiatives in the Global Young Academy, particularly with respect to what happens in the scientific career before the age of 40 and what will be seen later in their career development (Respondent PN4).

I believe that OWSD Guatemala National Chapter in its different work teams is working in a certain way on that since we are not replacing what a ministry or secretariat does at the government level, I believe that we are integrating ourselves as women to be able to work as a team on issues that we are passionate about and that is key, and it would be good to have international support for this (Respondent GT14).

#### Critical Perspective of Science Diplomacy

Many of the collaborations involving science diplomacy have been carried out without the explicit use of such terminology. Scientific and technological exchange is expanding and opening the opportunity for more collaborations in the region. Yet the lack of understanding of the concept of “science diplomacy” increases the risk of misunderstandings.

I was in charge of that for 10 years at PAHO working with international health, I think that in international health it is basic and it is essential to use science diplomacy tools, but I have problems with the term “diplomacy” next to science, it is like a qualifier, a deviation of the core (Participant SV6).

Mistrust in the links between politics and academia is a dilemma that needs to be considered: sometimes there is misalignment or conflicts of interest:

Science and politics are closely related. The downside is prioritizing one over the other. It has never seemed to me that the University of El Salvador prioritizes politics over science. Science can help El Salvador to make progress toward development. I think that these two fields can interact [science and policy], but you have to think about cooperation between sectors. Links need to be established with institutions and their support must be sought for any initiative, if there is no institutional support, no project can work (Participant SV7).

The predominance of the English language in international engagement might be a counterproductive component. For women in Science English language is mandatory if they want to continue their studies and research outside the Latin American region. Most scholarship applications and information are in English, available host institutes teach in English and vital publications are in English. Sometimes even a third language is needed. In Central America, where Spanish is the first language of most citizens, English is always at the very best a second language and is therefore a significant barrier for women who want to get access to higher level education.

With proficiency in English, it is possible to participate in conferences, publications and communicate the knowledge that has been produced, publish it internationally; you have to have that knowledge first and consolidate the language well to be able to do it. (Participant HN10).

## Conclusion and Implications

Central America faces pressing unbalanced female representation in science. The significant gaps the region faces in terms of science and technology capacities are also reflected in the limited experiences within the region of building communities among women scientists. While exceptional networks among women scientists have been reported, the gender perspective has been given marginal attention. Valuable initiatives were identified aiming at organizing groups of women scientists. However, some of these initiatives were episodic interventions with no sustainability. In other cases, well-intended leaders mobilized an important number of researchers to form communities, yet different factors impeded the communities to survive and faded over time. In other words, the few cases of community building among scientists in Guatemala, El Salvador, Honduras and Panama, are meaningful and provide lessons to understand why the existence of such communities is so limited and highlights the different barriers faced particularly by women researchers. In scientifically-lagging countries, the construction of communities of women scientists has special relevance as the number of well-trained researchers is still low and the actors involved in research ecosystems are still developing. Community building among women scientists in Central America offers an array of opportunities and benefits that can have a positive impact on their individual career development. More importantly, increasing the presence of women in science inspires children and young girls and reduces the pervasive gender gaps in science which are evident in these countries.

The women pioneering in community building in the scientific field have had to overcome several barriers at the individual, family, institutional and societal level. Most of those barriers remain present in their everyday activities such as: gender stereotypes, imbalances in the pursuit of family/professional life equilibrium and socially-assigned roles. Findings suggest there have not been systematic institutional policies at the national level to foster community building among women scientists in Central America. The existing examples of community building, although valuable have remained temporal and eventually have been discontinued. These outcomes are the result of numerous difficulties involving the unmet needs of nurturing leadership, connecting women in different moments of their careers (generational breaks) and the lack of resources. In addition, the challenges discussed in this article leave women scientists with very limited resources, energy and motivation to engage in community building exercises.

In this context, science diplomacy practices have played a key role in supporting incipient community building among women scientists in Central America and represent a helpful conceptual framework to shift the prevalent top-down paradigm toward a bottom-up approach. Some of the areas in which science diplomacy may incorporate further possibilities to encourage and support the participation of Central American women in communities include: mapping the scientific female diaspora, facilitating collaborative work with women in other regions, enable achievement of milestones in the career development of women, nurturing leadership, and mentorship, among others. Guatemala, El Salvador, and Honduras have relatively similar contexts, while Panama shows more advances in community building among women scientists. Specific actions have been taken by the Panama National Secretariat of Science and Technology producing focused reports on the participation of women in science (SENACYT, 2020a) and partnering with stakeholders from other sectors to provide a career development path for women in science (SENACYT, 2020b). In addition, Panama was the first country in Latin America with a national strategy in science diplomacy (SENACYT, 2020c), with the decisive involvement of the Ministry of Foreign Affairs and SENACYT, among other stakeholders of the science and technology ecosystem.

In summary, science diplomacy could incorporate further actions regarding community building among Central American women through the observation of principles such as inclusiveness, networking, and deliberation:

(1) Science in Diplomacy: this perspective could strengthen the conventional international engagement of Central American countries with further elements of capacity building through training with evidence-based educational programs in topics such as: intercultural relations, networking, effective communication, leadership, negotiation, empowerment, conflict mediation, emotional and social intelligence, science communication, languages, strengthen scientific capacities to female scientists. By strengthening these communities and networks a directory of female scientific advisors and specialists could be created. There should be a focus on addressing special challenges and for the creation of evidence-based policies from a bottom-up approach. Diplomacy for Science: this can be a tool for articulating why networks are necessary to strengthen the role and activities of female communities. For example, multi-actor involvement (i.e., academia, government, private sector, national academies of sciences and civil society) can create sustainable models and the win-win negotiation of equal access and opportunities for all participants. Diplomacy also plays an important role in identifying and grouping together national communities that have been established in the diaspora. This could help the establishment of new channels of cooperation and networking. Special consideration could be given to scientists working abroad and how they can enhance mutual and beneficial collaboration with those scientists still based in the home country. In a similar vein, training in diplomacy can help science negotiators obtain better conditions. For example, access to opportunities for engagement with national scientists and institutions should be encouraged for both those researchers based in the home country and those currently residing in the diaspora. Links between these scientists and regional and international organisms could be facilitated, with, for example, special relationships established between SICA, UNESCO and initiatives for women in science. In this way, other actions might be facilitated such as the multinational allocation and creation of projects for research, fostering scientific mobility, intra-regional focused activities, and the promotion of private investment for science. In all of these activities, it will be essential to have an underlying strategy of gender inclusion and awareness in those scientists who represent their nations at national and international levels.
(2) Science for Diplomacy: Central American community building can play an important role for diplomacy. For example, the uniqueness of the social and geographical conditions of the Central American region positions the territory as a natural laboratory for science. Through collaborations enhanced by communities with international peers and institutions not only can we enhance science locally and globally but also tighten relations with our traditional commercial and political partners while at the same time reducing the barriers with other countries in which there is a lack of adequate diplomatic relations. When scientists unite to study the common challenges in the region, this creates an ecosystem for gaining spaces in decision making and impacting societies at a national and international level through evidence-based culturally adapted mechanisms. Community Building in the post-pandemic world will provide a changing scenario with the use of different platforms for interactions. Numerous respondents referred to logistics, transportation, and other elements of physical mobility as obstacles for in-presence interactions among women scientists within their countries. With these recent experiences of on-line interactions, further exchanges and collaborations could be achieved.

## Data Availability

The raw data supporting the conclusions of this article will be made available by the authors, without undue reservation.

## Footnotes

1 RedCTI https://redcti.senacyt.gob.gt/portal/index.php

2 Acacienciasgt https://www.acacienciasgt.org/

3 REDISAL https://www.redisal.org.sv/newAdmin/directorio.php

4 Honduras Global http://hondurasglobal.org/

5 APANAC http://www.apanac.org.pa/

6 Ciencia en Panamá: https://www.cienciaenpanama.org/

7 ADEMCIT: https://ademcit.wordpress.com/2010/01/17/hola-mundo/

8 AMUS: https://www.asociaciondemujeresuniversitarias.com/

9 Red LIES https://www.laprensagrafica.com/elsalvador/USAID-lanza-Red-de-Mujeres-Lideres-en-Educacion-Superior-20171019-0127.html

10 OWSD Guatemala https://owsd.net/network/guatemala

11 OWSD Honduras Global https://www.owsd.net/network/honduras
