# Supplementary material for: Participation in Communities of Women Scientists in Central America: Implications From the Science Diplomacy Perspective
Source: Front Res Metr Anal. 2021 Jul 12;6:661508. doi: 10.3389/frma.2021.661508 (PMC8344979; doi:10.3389/frma.2021.661508)
Supplement: Supplementary file 1 [file DataSheet1.docx]

**Instrument: Semi-Structured Interview -** Participation in Communities of Women Scientists in Central America: Implications from the Science Diplomacy Perspective

Date: _______________ / Name of the Interviewer: _______________ Name of the Interviewee: ___________

Mechanism: ___________ (zoom, googlemeet, face-to-face, phone) Duration of the Interview: ______ (minutes)

**Part 1: Trajectory**

Question 1: In which area of knowledge do you specialize in and how did you decide to study it?

Question 2: As a woman, what has been your greatest challenge to study this area of knowledge and

develop professionally in her country?

Question 3: Would you like to share any additional information about your career as a researcher /

scientist?

**Part 2: Knowledge and experience in building communities of women scientists and researchers. Understanding the Community (Network) of Scientists as a group of women who are associated because their mutual interest is the achievement of previously agreed objectives and knowledge results related to the generation of knowledge through research using the scientific method.**

Question 4: From your experience, have there been initiatives to build scientific communities of women in your country?

-If the answer is positive, what have these experiences consisted of? What institutions or individuals participated in these initiatives? How long did these initiatives last? If they have not lasted long, why do you think these initiatives have not been sustained?

-If the first question is negative, why do you think that initiatives of this type have not been created?

Question 5: In your personal experience, have you been actively involved in building any community or communities of women scientists inside or outside your country? If your answer is positive, can you please give us the details?

**Part 3: Challenges in building communities of women scientists and researchers**

Question 6: What personal / professional limitations (obstacles, challenges, barriers) do you consider exist / existed to achieve concrete initiatives to build communities of women scientists in your country?

Question 7: What social constraints (with the rest of the community of scientists) exist / existed to build communities of women scientists in your country?

Question 8: What institutional limitations do you consider exist / existed to build communities of women scientists in your country?

Question 9: What limitations at the country level do you consider that exist / existed to build communities of women scientists in your country?

Question 10: What is the main barrier that exists for women to get involved in scientific communities at the national and / or international level?

Question 11: What obstacles do you consider that limit the participation of women in scientific networks / communities at the international level?

**Part 4: Opportunities in building communities of women scientists and researchers**

Question 12: Do you know of any national or international institution and / or group that has successfully promoted the participation of women in science?

- If this answer is positive, what have these mechanisms and / or practices consisted of

Question 13: Have you participated in any mentoring programs for women scientists?

If the answer is positive, what benefits do you consider it has brought to your professional career? If the answer is negative, what do you think is the reason why these programs do not exist or are not accessible?

**Part 5: Scientific Diplomacy and Building Communities of Women Scientists and Researchers**

Question 14: The Madrid Declaration on Scientific Diplomacy (2019) defines this term as: “A series of practices at the intersection between science, technology and foreign policy”. One of the focuses of this framework is Diplomacy for Science, which focuses primarily on facilitating international scientific and engineering collaborations. This can be achieved with top-down strategic priorities for research or with bottom-up collaboration between individual scientists and researchers^[[1]](#footnote-1)^. With this in mind, do you know of your country's experiences in cooperation with other countries or international entities with initiatives that seek to link scientists from their country within and outside the national territory?

- If the answer is positive, what experiences are they? Has it been specifically for women?

Question 15: According to the environment and reality of your country, do you consider that cooperation actions can be developed between countries to promote the development of scientific projects in which women actively participate?

- If the answer is positive, in what area could it be developed (academic / university, government, private company, personal)? why?

- If the answer is negative, why couldn't it be given?

**Part 6: Implications for the Organization of Women for Science for the Developing World (OWSD).**

Question 16: Have you heard about the OWSD?

- If the answer is yes, are you a member or would you consider being a member of your country's chapter? and because?

Question 17: What is the role that your country's OWSD could play in fostering the collaboration of women in the field of science diplomacy? What benefits, in your experience, could this network bring to the professional development of more women?

Question 18: To your knowledge, is there any similar study to the one you are being interviewed for?

Question 19: Would you suggest any name/names of potential interviewees who meet the criteria for

our study? Please help us with her/their information

**Closing:** Thank you very much for your kind participation in this interview. We have taken ___ minutes to complete the interview, do not hesitate to send me any additional information at your convenience to this email address [kleinsy@gmail.com](mailto:kleinsy@gmail.com)

1. (Lorenzo Melchor, Izaskun Lacunza and Ana Elorza. 2020. What is science diplomacy? In: S4D4C Online course in European scientific diplomacy, module 2, Vienna: S4D4C) [↑](#footnote-ref-1)
